# Racial and Economic Segregation Over the Life Course and Incident Hypertensive Disorders of Pregnancy Among Black Women in California

**DOI:** 10.1093/aje/kwad192

**Published:** 2023-09-28

**Authors:** Brittney Francis, Michelle Pearl, Cynthia Colen, Abigail Shoben, Shawnita Sealy-Jefferson

**Keywords:** Black, hypertensive disorders of pregnancy, life course, neighborhoods, segregation, structural racism

## Abstract

Black women in the United States have the highest incidence of hypertensive disorders of pregnancy (HDP) and are disproportionately burdened by its adverse sequalae, compared with women of all racial and ethnic groups. Segregation, a key driver of structural racism for Black families, can provide information critical to understanding these disparities. We examined the association between racial and economic segregation at 2 points and incident HDP using intergenerationally linked birth records of 45,204 Black California-born primiparous mothers (born 1982–1997) and their infants (born 1997–2011), with HDP ascertained from hospital discharge records. Women’s early childhood and adulthood neighborhoods were categorized as deprived, mixed, or privileged based on the Index of Concentration at the Extremes (a measure of concentrated racial and economic segregation), yielding 9 life-course trajectories. Women living in deprived neighborhoods at both time points experienced the highest odds of HDP (from mixed effect logistic regression, unadjusted odds ratio = 1.26, 95% confidence interval: 1.13, 1.40) compared with women living in privileged neighborhoods at both time points. All trajectories involving residence in a deprived neighborhood in early childhood or adulthood were associated with increased odds of HDP, whereas mixed-privileged and privileged-mixed trajectories were not. Future studies should assess the causal nature of these associations.

## Abbreviations

CI: confidence interval
HDP: hypertensive disorders of pregnancy
OR: odds ratio
ICE: Index of Concentration at the Extremes
LCSC: Life Course Social Context and Disparities in Birth Outcomes

Maternal morbidity and mortality are urgent issues in the United States. For more than a decade there have been increases in maternal mortality rates, despite reduction in rates in most countries around the world (1, 2). However, the increase in rates has not been proportional across racial groups (2, 3). Significant racial disparities in maternal mortality and morbidity rates in the United States have persisted over time, with Black women disproportionately bearing the burden (3, 4). Black women are at higher risk of developing dangerous pregnancy complications that increase their risk for mortality and severe morbidity (1, 5, 6), significantly contributing to their 3-fold risk of maternal death relative to White women (4, 5).

One such group of complications are hypertensive disorders of pregnancy (HDP). Currently, approximately 10% of pregnancies are affected by HDP, and that has been rising over the past few years (6–9). They are among the leading causes of maternal morbidity and mortality, directly accounting for 7% of maternal deaths and 31% of postpartum hospitalization readmissions (10, 11). Women who have experienced a HDP also have higher risk for postpartum hypertension, stroke, diabetes, and heart disease (12–14). Like the overall trend of maternal morbidity and mortality, Black women in the United States are disproportionately burdened by HDP and their adverse sequalae compared with women of all other racial, ethnic, and nativity groups (15–17). Between 2017 and 2019, the prevalence of HDP among Black women was 20.9%, followed by 16.4% for American Indian/Alaska Native women (18) compared with 14.7% for White women.

Researchers have been unable to explain these disparities after adjusting for traditional microlevel socioeconomic factors like education, income, and insurance status (19, 20). Similar to outcomes for infant-related outcomes with wide racial disparities, Black race—assumed to be a natural category based on alleged inherent racial biological differences—was considered a risk factor for HDP until very recently (19). There has been significant research documenting how the various forms of oppression and violence that Black women experience during pregnancy, rather than socially assigned race, can increase risk for adverse birth outcomes for Black infants, contributing to the disparities we see. However, these associations are critically understudied for HDP among Black women.

One of the most salient and longstanding forms of anti-Black racism in the United States is residential segregation (21, 22). Since the advent of slavery and legal racial discrimination in the United States up through the Civil Rights Act of 1964, Black people were forced to live in certain neighborhoods through legalized racist housing policies and practices such as redlining, racial covenants, and real-estate steering (22–24). Despite decades since the termination of legalized segregation, the United States remains very highly residentially segregated for racially minoritized people (25). Research shows that racialized residential segregation, especially when compounded with concentrated poverty, has adverse impacts on health by disproportionately exposing Black people to neighborhoods that: 1) are often underresourced and limit opportunities for upward socioeconomic mobility (26, 27), 2) have limited access to health-promoting goods and services (28, 29), and 3) overexpose them to harmful environmental pollutants (30–32). Additionally, these neighborhoods are also often targeted with other forms of marginalization (e.g., overpolicing, evictions, food deserts) that create stress and additional barriers to accessing resources and goods necessary for a healthy lifestyle (33, 34).

The association between residential segregation and HDP has been explored in the literature. One study examined the relationship between residential segregation and HDPs among Black women in Chicago between 2009 and 2013 and showed that residential segregation, especially the intersection of racial composition and neighborhood income levels, was associated with increased risk of experiencing a HDP among Black women (35). Specifically, they found that women who lived in areas that were predominantly Black and low income had the highest risk of developing a HDP (35). However, these associations have only been explored cross-sectionally (during pregnancy), never over the life course. This is important to explore because the life-course perspective highlights that similar experiences during different time points in life can have varying impacts (36, 37). It is recognized in studies of segregation that children and adults are affected differentially by segregation, with children experiencing additional impacts, such as educational inequality and exposures to environmental hazards during sensitive periods of development that can have future implications for pregnancy complications (38, 39).

To address this gap in the literature, this study explored the association between residential segregation at 2 sensitive key points over the life course in relation to reproductive health and incident HDP among Black women. To our knowledge, no prior study has explored the association between segregation over a woman’s life course and HDP.

## METHODS

### Study population and procedures

The present study uses a subset of a geocoded, intergenerational, population-based cohort created by the Life Course Social Context and Disparities in Birth Outcomes (LCSC) Study (40) from several sources. The California Biobank Program Linked Dataset (BLD) housed at the California Department of Public Health includes linked birth, fetal death, and death records with data from 2 statewide screening programs. The BLD intergenerationally linked births from 1982 to 2011. Singleton live birth records with an indication that the mother was born in California in or after 1982 were eligible for linkage to maternal birth certificate records (*n* = 98,464) (40).

Maternal information (sex, date of birth, and first, last, and maiden name) from birth certificates was used to create maternal-infant dyads and group births to the same women via a proprietary matching program using mathematical string-matching algorithms that model human decision making (Tibco Software Inc, Palo Alto, California), followed by programmed checks and extensive clerical review. Approximately 92% (*n* = 91,056) of the eligible birth certificates were successfully linked to the mother’s birth certificate (40). The LCSC study geocoded address data from maternal newborn-screening data and infant birth records to obtain census tract information from both time points (40) and linked to hospital discharge data for 2000–2011. Distributions of parity, education, age, insurance, and preterm birth were nearly identical for those eligible to link, those successfully linked, and those linked and successfully geocoded.

The final sample for analysis (*n* = 45,204) included first-time Black mothers who were not diagnosed with chronic hypertension prior to pregnancy and had complete geocoded address data from both birth certificates (see Web Tables 1–6, available at https://doi.org/10.1093/aje/kwad192, for sample derivation). The study was approved by the California Health and Human Services Agency Committee for the Protection of Human Subjects (Project # 14-01-1466) and Ohio State University’s Institutional Review Board (2020H0093).

### Exposure ascertainment

The Index of Concentration at the Extremes (ICE) (41–43) is an increasingly used multiscalar measure of residential segregation that classifies neighborhoods as deprived or privileged based on the racial and/or economic composition of residents within a determined geographical unit (44). For our study, we used the combined ICE to reflect racialized economic segregation. The ICE scale is a continuous score that ranges from −1 to 1, where −1 corresponds to a neighborhood that is completely composed of low-income Black residents (most deprived) and 1 corresponds to a neighborhood that is completely composed of high-income White residents (most privileged) (41, 43, 45). Although mathematically an ICE value of zero could indicate that a neighborhood either has no individuals or an equal number at the extremes, in actuality, as empirically demonstrated (41–43), US patterns of segregation render it highly implausible that an area such as a census tract would have an equal distribution of Black people at the lowest income levels and White people at the highest income levels (43–45). Studies show that neighborhoods comprised predominantly of low-income Black residents are more likely to experience underinvestment and be deprived of necessary healthy resources, while neighborhoods comprised predominantly of high-income and White residents receive significant investment in community infrastructure and are often located in close proximity to healthy resources (46, 47). ICE scores were developed in the LCSC Study at the census tract level for all women at the time of their own births and first pregnancies, as part of a prior study (48). Tracts were derived from geocoded addresses corresponding to the time of a woman’s birth and the time of her first pregnancy. ICE scores were calculated using data from the decennial census closest to each year of birth as follows: Within each tract, the number of non-Hispanic Black persons with household income below the 20th income percentile was subtracted from the number of non-Hispanic White persons with annual household income at or above the 80th percentile, then divided by the total population in the tract. Additional details on ICE measure calculation have been previously published (41, 42).

We developed a novel segregation-mobility exposure variable for this analysis based on geocoded addresses at 2 sensitive time points related to the reproductive life course: time of the pregnant person’s birth and the time of delivery for their offspring. We utilized these data to reflect early-childhood neighborhood context and adulthood neighborhood context, as well as neighborhood change between those time periods. For each time point, tertile cutpoints of ICE scores were used to classify women’s census tracts as deprived (lowest tertile), mixed (middle tertile), or privileged (highest tertile) neighborhoods (see Web Tables). The final segregation-mobility variable was categorical in nature and had 9 categories: 1) childhood and adulthood privilege (life-course privilege), 2) childhood privilege and adulthood mixed, 3) childhood privilege and adulthood deprived, 4) childhood mixed and adulthood privileged, 5) childhood and adulthood mixed, 6) childhood mixed and adulthood deprived, 7) childhood deprived and adulthood privilege, 8) childhood deprived and adulthood mixed and 9) childhood deprived and adulthood deprived (life-course deprivation), representing all potential combinations of the proxied childhood and adulthood neighborhoods. Women living in privileged neighborhoods over the life-course were the referent group.

### Outcome ascertainment

The outcome of interest was an incident HDP. HDP in our sample was defined as a new diagnosis of gestational hypertension, pre-eclampsia, or eclampsia (49–51). HDP status was ascertained from linked hospital-discharge records. The final HDP variable was modeled as a binary variable (yes/no).

### Statistical approach

Descriptive statistics for available sociodemographic variables and other known risk factors for HDP were used to assess sample characteristics and their relationship with HDP. Correlations between childhood and adulthood ICE scores were assessed, using Pearson’s correlation coefficient. We used a mixed effect logistic regression model to account for clustering of individuals at the census tract level based on the addresses of the women during pregnancy. This type of modeling is recommended for examining neighborhood level associations with individual health in social epidemiology studies (52). All analyses were completed using STATA IC, version 16.0 (StataCorp LLC, College Station, Texas).

## RESULTS

The sociodemographic and behavioral characteristics for our final sample of 45,204 are shown in Table 1. The majority of women were between 18 and 22 years of age (65%, *n* = 29,359) and had at least completed high school (66%, *n* = 29,699). Most were born between 1982 and 1992 and gave birth between 2007 and 2011. Approximately 11% of the sample (*n* = 4,908) was diagnosed with HDP: 5% had gestational hypertension (*n* = 2,272), 5.8% had pre-eclampsia (*n* = 2,615), and 0.3% had eclampsia (*n* = 117). ICE scores in childhood ranged from −0.39 to 0.43, with a median and mean score of −0.02 in the overall sample. ICE scores during adulthood ranged from −0.25 to 0.46, with a median and mean score of −0.01 in the overall sample. Childhood and adulthood ICE scores were weakly correlated (ρ = 0.25). Approximately 44% of the women gave birth while living in a census tract with the same neighborhood classification category (deprived, mixed, privileged) as their childhood neighborhood. Twenty-nine percent of the women gave birth while living in a census tract that was less privileged than the census tract of their childhood (downward mobility), and 27% gave birth while living in a census tract that was more privileged than the census tract of their childhood (upward mobility). The most prevalent individual category was life-course deprivation (16%, *n* = 7,335), followed by the life-course privilege category (15%, *n* = 6,911). Distributions of ICE scores by participant characteristics are shown at both time points in the Web Tables.

**Table 1:** Characteristics of and Association With Hypertensive Disorders of Pregnancy Among Black Women in California, 1982–2011

**Characteristic**	**Total Sample**		**No HDP**		**HDP**		**OR**	**95% CI**
	**No.**	**%**	**No.**	**%**	**No.**	**%**		
Total	45,204	100	40,296	91	4,908	11.0		
Age, years								
<18	9,165	20.3	8,174	20.3	991	20.2	1.00	Referent
18–22	29,359	65.0	26,223	65.1	3,136	63.9	0.99	0.91, 1.06
23–29	6,680	14.7	5,899	14.6	781	15.9	1.09	0.99,1.21
Education								
Less than high school graduation	14,562	32.2	12,956	32.2	1,606	32.7	1.00	Referent
High school graduation	17,592	38.9	15,677	38.9	1,915	39.0	0.99	0.92, 1.06
Beyond high school	12,107	26.8	10,829	26.8	1278	26.0	0.95	0.88, 1.03
Missing	943	2.1	834	2.1	109	2.2		

Abbreviations: CI, confidence interval; HDP, hypertensive disorders of pregnancy; OR, odds ratio.

**Figure 1:**
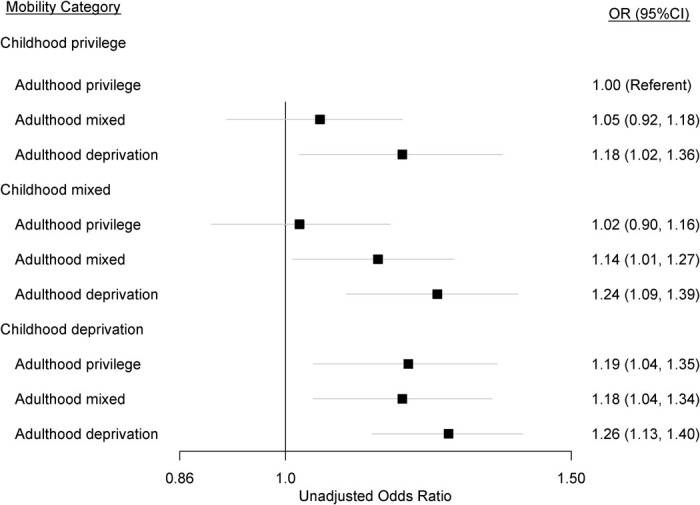
Odds ratios (ORs) and 95% confidence intervals (CIs) from mixed effects logistic regression for life-course segregation mobility and hypertensive disorders of pregnancy among black women in California, 1982–2011.

In an unadjusted model, all but 2 trajectories were associated with increased odds of HDP compared with living in privileged neighborhoods over the life course (Table 2). Compared with life-course privilege, living in deprived neighborhoods over the life course was associated with the highest odds of having HDP (odds ratio (OR) = 1.26, confidence interval (CI): 1.13, 1.40), followed by living in a mixed neighborhood during childhood and deprived neighborhood during adulthood (OR = 1.24, CI: 1.09, 1.39). A clear stepwise increase in odds ratios of HDP relative to life-course privilege is evident for women who lived in privileged or mixed neighborhood during childhood, as they move from privilege to deprived neighborhoods in adulthood (Figure 1). However, this stepwise trend is not seen among women who lived in deprived neighborhoods during childhood. ORs and CIs of experiencing an incident HDP relative to life-course privilege were similar for women who lived in deprived neighborhoods during childhood but then lived in privileged (OR = 1.19, CI: 1.04, 1.34), mixed (OR = 1.18, CI: 1.04, 1.34), or deprived (OR = 1.26, CI: 1.13, 1.40) neighborhoods during adulthood. Notably, women who lived in a deprived neighborhood during childhood and lived in a privileged neighborhood during adulthood (fully upwardly mobile) had similar ORs for HDP relative to life-course privilege as women who lived in a privileged neighborhood during childhood and gave birth while living in a deprived neighborhood (fully downwardly mobile) Adjusting for age and education attenuated estimates slightly (data not shown).

**Table 2:** Odds Ratios and 95% Confidence Intervals for the Association Between Life-Course Segregation Mobility and Hypertensive Disorders of Pregnancy Among Black Women in California, 1982–2011

**Mobility Category**	**No.**	**% HDP**	**Unadjusted**	
			**OR**	**95% CI**
Childhood privilege				
Adulthood privilege	6,911	9.7	1.00	Referent
Adulthood mixed	5,340	10.1	1.05	0.92, 1.18
Adulthood deprivation	2,810	11.2	1.18	1.02, 1.36
Childhood mixed				
Adulthood privilege	4,574	9.9	1.02	0.90, 1.16
Adulthood mixed	5,554	10.9	1.14	1.01, 1.27
Adulthood deprivation	4,944	11.7	1.24	1.09, 1.39
Childhood deprivation				
Adulthood privilege	3,583	11.3	1.19	1.04, 1.35
Adulthood mixed	4,153	11.3	1.18	1.04, 1.34
Adulthood deprivation	7,335	11.9	1.26	1.13, 1.40

Abbreviations: CI, confidence interval; HDP, hypertensive disorders of pregnancy; OR, odds ratio.

## DISCUSSION

In our analysis of life-course neighborhood racialized economic segregation among Black women, most trajectories were associated with higher odds of HDP compared with living in neighborhoods of privilege over their life course. In particular, trajectories including residence in a deprived neighborhood at either time point were associated with increased odds of HDP, whereas mixed-privileged and privileged-mixed trajectories were not. Living in deprived neighborhoods at both time points was associated with the highest odds of HDP compared with living in privileged neighborhoods over the life course.

One interesting and important finding is that upward socioeconomic mobility (living in a more privileged neighborhood in adulthood than in childhood) was not associated with increased risk of HDP among Black women who grew up in mixed neighborhoods, but it is associated with increased risk of HDP among Black women who grew up in deprived neighborhoods, compared with women who lived in privilege at both time points. Clear stepwise gradients, whereby the likelihood of experiencing HDP increases as their adulthood neighborhood worsened, were evident among women who lived in mixed and privileged neighborhoods during childhood. However, this stepwise gradient is not evident among Black women who were residents of deprived neighborhoods during childhood. Regardless of the neighborhood they resided in during adulthood, their risk of HDP did not substantially differ. These results are particularly notable for 3 overarching reasons. First, they point to the importance and enduring impact of childhood residence in addition to adulthood residence. Specifically, childhood residence in deprived neighborhoods may have long-lasting effects that should be explored. Second, the results suggest that Black women who grew up in deprived neighborhoods may not experience the same health benefits typically attributable to upward social mobility (53–55). As such, it is vital to study and address neighborhood inequalities during early life as potential life-course risk factors for HDP. Third, the results suggest that living in a deprived neighborhood during childhood may partially explain the increased risk of HDP among Black women and is a potential pathway through which racial disparities in health are socially passed from one generation to the next, even among Black women of higher socioeconomic position. The finding of increased HDP risk for those living in deprived neighborhoods, regardless of privileged or mixed neighborhoods during childhood, emphasizes the importance of understanding adulthood neighborhood environments in relation to Black women’s health. Taken together, the study findings suggest that equitable neighborhood opportunities and resources for both children and adults are necessary for improving health equity.

Collectively, these findings are consistent with the sociological and epidemiologic literature suggesting that impacts of segregation should be explored over the life course (36, 56). During childhood, living in a highly segregated and underresourced neighborhood is associated with the quality and years of education children receive (57, 58), the risk of exposure to environmental toxins during key developmental periods (59), increased exposure to smoking advertisements (60, 61), and reduced access to fresh food (56, 62). All of these factors have long-term impacts on adult health, including pregnancy. During adulthood, living in a highly segregated and underresourced neighborhood is associated with limited job opportunities (63), longer commutes on public transportation (64), limited access to health-care providers (65), increased exposure to deadly interactions with police officers (66), reduced access to fresh and nutritious foods (28), and increased stress due to neighborhood conditions (67). This study points to the need to explore residential history over the life course and the differential temporal pathways through which segregation may affect HDP and other pregnancy complications to further understand the life-course impacts of structural racism on health.

This study had a few limitations that should be taken into consideration. First, data on HDP came from hospital discharge data, which could result in missed cases of HDP and misclassification bias. However, an internal cross check at the California Department of Health against maternal medical records found 97% sensitivity for HDP from hospital discharge data among Black women and did not vary by neighborhood type. Secondly, the study’s conclusions are limited to associations. These associations may support future causal analysis in studies with richer covariate information, and the ability to distinguish mediating pathways through health behaviors. Such analyses should consider that a reduction in segregation may also decrease chronic hypertension (excluded in this study), and thereby increase the number of women susceptible to incident HDP. Finally, based on the timeframe of the linkages (1982–2011), the maximum age possible in the sample was 29. Although only 18% of Black women delivering their first infant in California in 2011 were age 30 or older, older women are at increased risk of HDP (18). Including older women could affect the distribution of the trajectories and allow for more upward mobility. Future studies should explore this association among women of a wider age range.

The study also had several strengths. Primarily, it is the first study, to our knowledge, to explore the relationship between residential segregation over the life course and HDP. The results from this study highlight the need for epidemiologic studies that examine neighborhoods both prior to and during pregnancy to understand the variable and temporal impacts of segregated neighborhoods over the life course. Second, the linking of several statewide data sources allowed us to build life-course trajectories and examine 2 critical time periods over the life course for pregnancy outcomes. The analysis showed that women can live in the same types of neighborhoods during pregnancy but have differential risk of HDP due to early-life exposure. Future studies should explore additional meaningful time points over the life course. Finally, our intracategorical sample allowed us to examine the differential impact of neighborhood quality among Black women. Such an analysis requires a large sample size, and the California data set includes the largest sample of Black women with cross-generational neighborhood information in the country. An intracategorical analysis also contributes to dispelling the myth that Black race itself should be used as a clinical risk factor, by highlighting heterogeneity among Black women and the important role of life-course neighborhoods. Risk assessment should address other structural factors that can have biological and behavioral impacts on the risk of developing HDP among Black women, creating the disparities we see.

An environmental oppression framework posits that residential environments composed predominantly of low-income and racially marginalized people are harmed by various inequitable policies and practices that create adverse social, built, and natural environments (33, 68, 69). Since one’s residential environment is linked to key resources needed to create and maintain a health-promoting lifestyle in our society (69), living in a neighborhood where resources are consistently extracted, exploited, or underfunded (deprived neighborhoods) can adversely affect health outcomes for the people living there. For example, neighborhood-level inequities in infrastructure and access to fresh and nutritious foods directly affect Black women’s ability to implement key individual-level prevention and management interventions and recommendations for HDP, such as diet and exercise. Exploring the complex impact of segregation as a fundamental cause of inequitable outcomes in hypertensive disorders of pregnancy is necessary given the current maternal morbidity and mortality crisis among Black women. The findings of this research suggest that equitable resources in all neighborhoods and for all residents, such that women do not live in a deprived neighborhood during their life course, may serve as a strong population health intervention for hypertensive disorders of pregnancy and creating equity for Black women.

## Supplementary Material

Web_Material_kwad192
