# Supplementary material for: Racial and Economic Segregation Over the Life Course and Incident Hypertensive Disorders of Pregnancy Among Black Women in California
Source: Am J Epidemiol. 2023 Sep 28;193(2):277–84. doi: 10.1093/aje/kwad192 (PMC11031219; doi:10.1093/aje/kwad192)
Supplement: Web_Material_kwad192 [file web_material_kwad192.pdf]

Web Material

Racial and Economic Segregation Over the Life Course and Incident Hypertensive Disorders of Pregnancy Among Black Women in California

Brittney Francis, PhD, MPH; Michelle Pearl, PhD, MPH; Cynthia Colen, PhD, MPH; Abigail Shoben, PhD, MS; Shawnita Sealy-Jefferson, PhD, MPH

Table of Contents: Web Tables 1–6

Web Table 1: Sample derivation

Exclusions	Included	Excluded
1. Black and born in CA during newborn screening time period	98,464	n/a
2. Linked to mother birth record	91,056	7,408
3. Linked to mother NBS address	87,201	3,855
4. Good mother geocode	78,295	8,906
5. Good child geocode	74,686	3,609
7. First birth (parity=0)	47,913	26,773
8. Linked to hospital discharge data	46,614	1,299
9. Drop chronic hypertension	46,118	496
6. Drop if missing outcome or exposure data	45,204	914

Web Table 2: Distribution and Cutpoints of Childhood ICE scores for mobility tertiles Among Black Women in California, 1982-2011

	N	Min	Med	Max
Deprived	15,071	-0.39	-0.07	-0.05
Mixed	15,072	-0.05	-0.02	0.00
Privilege	15,061	0.00	0.03	0.43

Web Table 3: Distribution and Cutpoints of Adulthood ICE scores for mobility tertiles

Among Black Women in California, 1982-2011

	N	Min	Med	Max
Deprived	15,089	-0.25	-0.06	-0.03
Mixed	15,047	-0.03	-0.01	0.01
Privilege	15,068	0.01	0.03	0.46

Web Table 4. Distribution of participant characteristics by childhood ICE tertile

		Tertile Categories of Childhood ICE Score		
**Demographic Characteristics**	**Levels**	**Dep (n=15,701)**	**Int (n=15,072)**	**Priv (n=15,061)**
Age (years)	<18	3,087 (20.5)	2,841 (18.9)	3,237 (21.5)
	18-22	9,813 (65.1)	9,774 (65.9)	9,772 (64.9)
	23-29	2,171 (14.4)	2.457 (16.3)	2,052 (13.6)
BMI	Normal	4,145 (31.1)	4,111 (27.3)	5,066 (33.6)
	Overweight	1,777 (32.2)	1,726 (11.5)	2,023 (13.4)
	Obese	1,503 (31.6)	1,552 (10.3)	1,705 (11.3)
	Missing	7,646 (50.7)	7,683 (51.0)	6,267 (41.6)
Education	Less than High School	5,194 (34.5)	4,628 (30.7)	4,740 (31.5)
	High school	5,947 (39.5)	5,980 (39.7)	5,665 (37.6)
	More than high school	3,632 (24.1)	4,154 (27.5)	4,321 (28.7)
	Missing	298 (1.9)	310 (2.1)	335 (2.2)
Any smoking	No	7,672 (50.9)	7,731 (51.3)	9,019 (59.9)
	Yes	233 (1.5)	325 (2.2)	415 (2.8)
	Missing	7,166 (47.6)	7,016 (46.5)	5,627 (37.4)
PIHD	No	13,323 (88.4)	13,436 (89.1)	13,537 (89.9)
	Yes	1,748 (11.6)	1,636 (10.9)	1,524 (10.1)

Web Table 5. Distribution of participant characteristics by adulthood ICE tertile

		**Tertile Categories of Adulthood ICE Score**		
**Demographic Characteristics**	**Levels**	**Dep (n=15,089)**	**Int (n=15,047)**	**Priv (n=15,068)**
Age (years)	<18	3,381 (22.4)	3,035 (20.2)	2,749 (18.2)
	18-22	9,788 (64.9)	9,892 (65.7)	9,679 (64.2)
	23-29	1,920 (12.3)	2,120 (14.1)	2,640 (17.5)
BMI	Normal	4,028 (26.7)	4,701 (31.2)	4,593 (30.5)
	Overweight	1,703 (11.3)	1,908 (12.7)	1,915 (12.7)
	Obese	1,444 (9.6)	1,695 (11.3)	1,621 (10.8)
	Missing	7,914 (52.4)	6,743 (44.8)	6,939 (46.0)
Education	Less than High School	5,442 (36.1)	5,031 (33.4)	4,089 (27.1)
	High school	5,938 (39.4)	5,870 (39.0)	5,784 (38.4)
	More than high school	3,434 (22.8)	3,828 (25.44)	4,845 (32.1)
	Missing	275 (1.8)	318 (2.1)	350 (2.3)
Any smoking	No	7.383 (48.9)	8,734 (58.0)	8,305 (55.1)
	Yes	231 (1.5)	384 (2.6)	358 (2.4)
	Missing	7,475 (49.5)	5,929 (39.4)	6,405 (42.5)
PIHD	No	13,320 (88.3)	13,436 (89.3)	13,540 (89.9)
	Yes	1,769 (11.7)	1,611 (10.7)	1,528 (10.1)

Web Table 6: Distribution of participant characteristics by social mobility category

		Mobility Categories								
**Demographic Characteristics**	**Levels**	**CP/AP**	**CP/AI**	**CP/AD**	**CI/AP**	**CI/AI**	**CI/AD**	**CD/AP**	**CD/AI**	**CD/AD**
Age (years)	<18	1,329 (19.2)	1,239 (23.2)	669 (23.8)	760 (16.6)	1,027 (18.5)	1,054 (21.3)	660 (18.4)	769 (18.5)	1,658 (22.6)
	18-22	4,464 (64.6)	3,475 (65.1)	1,833 (65.2)	2,906 (63.5)	3,648 (65.7)	3,220 (65.1)	2,309 (64.4)	2,769 (66.7)	4,735 (64.6)
	23-29	1,118 (16.1)	626 (11.7)	308 (11.0)	908 (19.9)	879 (15.8)	670 (13.6)	614 (17.1)	615 (14.8)	942 (12.8)
BMI	Normal	2,287 (33.1)	1,904 (35.7)	875 (31.1)	1,280 (28.0)	1,602 (28.8)	1,229 (24.9)	1,026 (28.6)	1,195 (28.8)	1,924 (26.2)
	Overweight	914 (13.2)	727 (13.6)	382 (13.6)	544 (11.9)	632 (11.4)	550 (11.1)	457 (12.8)	549 (13.2)	771 (10.5)
	Obese	784 (11.3)	623 (11.7)	298 (10.6)	479 (10.5)	628 (11.3)	445 (9.0)	358 (10.0)	444 (10.7)	701 (9.6)
	Missing	2,926 (42.3)	2,086 (39.1)	1,255 (44.7)	2,271 (49.7)	2,692 (48.5)	2,720 (55.0)	1,742 (48.6)	1,965 (47.3)	3,939 (53.7)
Education	Less than High School	1,842 (26.7)	1,880 (35.2)	1,018 (36.2)	1,216 (26.6)	1,734 (31.2)	1,678 (33.9)	1,031 (28.8)	1,417 (34.1)	2,746 (37.4)
	High school	2,631 (38.1)	1,975 (37.0)	1,059 (37.7)	1,773 (38.8)	2,248 (40.5)	1,959 (39.6)	1,380 (38.5)	1,647 (40.7)	2,920 (39.8)
	More than high school	2,265 (32.8)	1,376 (25.8)	680 (24.2)	1,494 (32.7)	1,442 (26.0)	1,218 (24.6)	1,086 (30.3)	1,010 (24.3)	1,536 (20.9)
	Missing	173 (2.5)	109 (2.0)	53 (1.9)	91 (2.0)	130 (2.3)	89 (1.8)	86 (2.4)	79 (1.9)	133 (1.8)
Any smoking	No	4,065 (58.8)	3,367 (63.0)	1,587 (56.5)	2,356 (51.5)	3,082 (55.5)	2,293 (46.4)	1,884 (52.6)	2,285 (55.0)	3,503 (47.8)
	Yes	171 (2.5)	174 (3.3)	70 (2.5)	116 (2.5)	125 (2.2)	84 (1.7)	71 (2.0)	85 (2.1)	77 (1.0)
	Missing	2,675 (38.7)	1,799 (33.7)	1,153 (41.0)	2,102 (46.0)	2,347 (42.3)	2,567 (51.9)	1,628 (45.4)	1,783 (42.9)	3,755 (51.2)
PIHD	No	6,241 (90.3)	4,801 (89.9)	2,495 (88.8)	4,121 (90.1)	4,950 (89.1)	4,365 (88.3)	3,178 (88.7)	3,685 (88.7)	6,460 (88.1)
	Yes	670 (9.7)	539 (10.1)	315 (11.2)	453 (9.9)	604 (10.9)	579 (11.7)	405 (11.3)	468 (11.3)	875 (11.9)
